# The Role of Biliary Carcinoembryonic Antigen-Related Cellular Adhesion Molecule 6 (CEACAM6) as a Biomarker in Cholangiocarcinoma

**DOI:** 10.1371/journal.pone.0150195

**Published:** 2016-03-14

**Authors:** J. Bart Rose, Camilo Correa-Gallego, Yu Li, James Nelson, Adnan Alseidi, W. Scott Helton, Peter J. Allen, Michael I. D’Angelica, Ronald P. DeMatteo, Yuman Fong, T. Peter Kingham, Kris V. Kowdley, William R. Jarnagin, Flavio G. Rocha

**Affiliations:** 1 Section of General, Thoracic and Vascular Surgery, Department of Surgery, Virginia Mason Medical Center, Seattle, Washington, United States of America; 2 Benaroya Research Institute, Seattle, Washington, United States of America; 3 Hepatopancreatobiliary Service, Department of Surgery, Memorial Sloan-Kettering Cancer Center, New York, New York, United States of America; 4 Department of Surgery, City of Hope Cancer Center, Duarte, California, United States of America; Texas A&M Health Science Center, UNITED STATES

## Abstract

**Objective:**

The aim of the present study is to determine if CEACAM6 can be detected in the bile of patients with biliary cancer and can serve as a diagnostic biomarker for cholangiocarcinoma.

**Summary Background Data:**

Distinguishing bile duct carcinoma from other diagnoses is often difficult using endoscopic or percutaneous techniques. The cell surface protein CEACAM6 is over-expressed in many gastrointestinal cancers and may be selectively elevated in biliary adenocarcinoma.

**Methods:**

Bile from patients with benign biliary disease and cholangiocarcinoma (hilar, intrahepatic and distal) was collected at the time of index operation. The concentration of CEACAM6 was quantified by sandwich enzyme-linked immunosorbent assay (ELISA) and correlated to pathologic diagnosis. Diagnostic capability of CEACAM6 was evaluated by Wilcoxon rank-sum, linear regression, multiple regression, and receiver operating characteristic (ROC) curve analysis.

**Results:**

Bile from 83 patients was analyzed: 42 with benign disease and 41 with cholangiocarcinoma. Patients in the benign cohort were younger, predominantly female, and had lower median biliary CEACAM6 levels than patients in the malignant cohort (7.5 ng/ml vs. 40 ng/ml; p = <.001). ROC curve analysis determined CEACAM6 to be a positive predictor cholangiocarcinoma with a CEACAM6 level >14 ng/ml associated with 87.5% sensitivity, 69.1% specificity, and a likelihood ratio of 2.8 (AUC 0.74). Multiple regression analysis suggested elevated alkaline phosphatase and the presence of biliary endoprostheses may influence CEACAM6 levels.

**Conclusion:**

Biliary CEACAM6 can identify patients with extrahepatic cholangiocarcinoma with a high degree of sensitivity and should be investigated further as a potential screening tool.

## Introduction

Biliary tract cancers have universally poor outcomes, with surgical resection offering the only chance for cure. If resected, 5 year survival rates of 30–40% for intrahepatic cholangiocarcinoma (IHCC) and 50% for extrahepatic cholangiocarcinoma (EHCC) can be achieved.[1–3] However, in unresectable disease the median overall survival is only 6–12 months.[4] Unfortunately, most tumors are discovered at an advanced stage due to a delay in diagnosis with only 30% of tumors found to be resectable at time of presentation.[1] While most bile duct carcinomas develop sporadically, there are some known risk factors, including parasitic infestation, choledochal cysts, hepatitis C virus, intrahepatic lithiasis, abnormal pancreatobiliary junction, thorotrast, and primary sclerosing cholangitis (PSC). PSC carries the highest risk, with a rate of cholangiocarcinoma development of 0.6% per year. Half of patients with PSC that develop cancer will do so within a year of diagnosis with a prevalence of 30–42%.[5,6]

Currently there are no biomarkers with sufficient sensitivity and specificity that can reliably screen high risk individuals or confirm the presence of malignancy in biliary strictures. The most widely utilized clinical biomarker for cholangiocarcinoma is serum carbohydrate antigen 19–9 (CA19-9). However, CA19-9 may also be elevated in pancreatitis, cholangitis, primary biliary cirrhosis, and with heavy tobacco use.[7] The range of sensitivity and specificity for CA19-9 has been shown to be between 53–92% and 50–98% depending on the cutoff value used and population studied.[8] Additionally, up to 10% of patients do not express the blood type Lewis antigen and therefore do not express CA19-9.[9,10] Alternative biomarkers have been identified and investigated, but to date there is no validated biomarker with clinically useful diagnostic capabilities for cholangiocarcinoma. Bile has been investigated as a potential source of diagnostic biomarkers as it is the most proximal fluid in direct contact with the malignant cholangiocyte. Proteomic analysis of human bile has identified a number of novel biomarker candidates, amongst them carcinoembryonic antigen-related cellular adhesion molecule 6 (CEACAM6).[11] Subsequent analysis by the same investigators suggested biliary CEACAM6 can differentiate malignant strictures from benign disease with high accuracy, but was limited by small numbers and heterogeneity of its malignant cohort.[12]

The cell-surface molecule CEACAM6 is a 90 kDa glycoprotein with seven extracellular domains, a glycosylphosphatidylinositol (GPI) anchor, and lacks an intracellular signaling domain.[13] It is a member of the immunoglobulin cell adhesion molecule superfamily, of which carcinoembryonic antigen (CEACAM5) is also a member. CEACAM6 is located predominately on the cell surface of gastrointestinal, respiratory and mammary epithelium and neutrophils where it plays a role in cellular adhesion.[14–16] CEACAM6 has been found to be overexpressed in colorectal cancer, pancreatic cancer, and IHCC, It is detectible in all stages of disease including hyperplastic polyps and adenomas, and may be associated with poor prognosis.[17–22] CEACAM6 appears to play a large part in regulating anoikis via a Akt/c-src signaling pathway and may regulate gemcitabine resistance in IHCC and pancreatic cancer.[22–25] The cell surface location on biliary epithelium makes CEACAM6 an ideal candidate for a bile-based biomarker for cholangiocarcinoma.

This study investigates the hypothesis that CEACAM6 concentrations in bile may be a biomarker of malignant disease, with the potential to differentiate cholangiocarcinoma from benign biliary disease.

## Methods

### Sample Collection

This project was reviewed and approved by the Virginia Mason IRB under identifier IRB12055. Written consent was obtained before sample collection. All patients undergoing resection for confirmed or suspected biliary cancer or benign biliary disease (cholelithiasis, cholecystitis, and bile duct stricture or injury) who were over 18 years of age and non-pregnant were eligible for bile collection at two tertiary care academic institutions specializing in hepatopancreatobiliary surgery (Memorial Sloan-Kettering Cancer Center and Virginia Mason Medical Center). Bile samples were collected between September 2010 and June 2013 from the bile duct of patients at time of operation. Samples were immediately vortexed, aliquoted, and snap frozen at -80°C until further analysis.

### Bile Analysis

CEACAM6 concentrations were determined by a commercial enzyme-linked immunosorbent assay (SEK10823, Sino Biological, Beijing, China). Bile was diluted ten-fold in sample buffer and analyzed in duplicate. Samples were incubated on plates coated with provided capture antibody, washed, and further incubated with provided detection antibody. Quantification was accomplished using provided streptavidin horseradish peroxidase conjugate and chemiluminescent substrate (Amersham #RPN-2109). Total bile salt concentrations were determined by 3-α-hydroxysteroid dehydrogenase (3-α-HSD) analysis and utilized as a surrogate for bile concentration.[26]

### Western Blot Analysis

Western Blot analysis was used to confirm specificity of the detection antibody provided in the ELISA kit. Samples were heat denatured, separated by 4–20% SDS-PAGE gel (Bio-Rad #456–1095), and transferred to 0.45 μm nitrocellulose membrane. The primary antibody was the biotinylated CEACAM6 detection antibody provided in the ELISA kit (1.5 μg/ml final concentration). Final detection was accomplished using the ELISA provided streptavidin horseradish peroxidase conjugate (1:2000 final dilution) and chemiluminescent substrate (Amersham #RPN-2109).

### Clinical Information

Patient demographics, preoperative biliary stent information and laboratory values (alkaline phosphatase, total bilirubin, and serum carcinoembryonic antigen and carbohydrate antigen 19–9 levels), and final pathologic diagnosis were abstracted from a prospectively maintained database of enrolled patients. The definition of an elevated CA19-9 level was set at > 100 U/mL based on accepted clinical standards. [27] An elevated Total Bilirubin was set at > 3 mg/dL based on the likelihood of clinically apparent jaundice. An elevated Alkaline Phosphatase was set at > 140 IU/L based on the upper limit of normal reported at our institutions.

### Statistics

Categorical variables were compared by Chi-squared or Kruskal-Wallis tests, while continuous variables were compared by Mann-Whitney rank sum. Sensitivity and specificity was determined using receiver operating characteristic (ROC) curves. To determine if various clinicopathologic parameters may be influencing CEACAM6 expression, both linear and multiple regression analyses were performed with log transformed CEACAM6 level as the dependent variable. Statistical analysis performed with MedCalc 14.10.2 Software (Mariakerke, Belgium). Statistically significant differences were defined as those with a *P* value of <.05.

## Results

### Baseline Demographics and Laboratory Values

Bile samples were collected from 83 patients, 42 with benign biliary disease (14 cholecystitis, 14 benign hepatobiliary tumors, 8 gallstone pancreatitis, 4 choledochal cysts, and 2 benign strictures) and 41 with biliary cancer. The cholangiocarcinoma cohort included 17 intrahepatic and 24 extrahepatic tumors. Age, gender, presence of a biliary endoprosthesis, biliary salt concentration, and serum concentrations of carbohydrate antigen 19–9 (CA19-9), carcinoembryonic antigen (CEA), alkaline phosphatase (ALP), and total bilirubin (T.Bili) are compared in Table 1. Complete pathologic staging is available in Table in S1 Table. The benign, IHCC, and EHCC cohorts were significantly different with respect to age at presentation (54.0 [41.0–60.0] vs. 65.0 [61.8–74.0] vs. 66.0 [57.5–75.5] years; p = <.001) and frequency of biliary stenting (12% vs. 6% vs. 83%; p = <.001). The three cohorts differed with respect to median levels of bile salts (8.4 [6.5–14.7] vs. 9.9 [5.7–20.4] vs. 4.9 [0.1–8.5] μmol/mL; p = .003), serum CA19-9 (18.0 [9.5–50.0] vs. 47.5 [26.0–199.3] vs. 181.0 [122.0–342.0] U/mL; p = .021), serum ALP (81.0 [68.3–105.5] vs. 98.0 [73.8–132.3] vs. 229.0 [149.0–564.0] IU/mL; p = <.001), and serum T.Bili (0.5 [0.4–1.0] vs. 0.6 [0.6–0.7] vs. 1.5 [0.6–3.5] mg/dL; p = <.001).

**Table 1:** Basic demographics of patients with benign and malignant disease.

Characteristic	Benign (n = 42)	Intrahepatic (n = 17)	Extrahepatic (n = 24)	*P* value
Age, yrs	54.0 (41.0–60.0)	65.0 (61.8–74.0)	66.0 (57.5–75.5)	<.001
Male, n (%)	15 (36)	8 (47)	14 (58)	.200
Stent, n (%)	5 (12)	1 (6)	20 (83)	<.001
Biliary salt concentration, umol/mL	8.4 (6.5–14.7)	9.9 (5.7–20.4)	4.9 (0.1–8.5)	.003
Alkaline Phosphatase (ALP), IU/L^1^	81.0 (68.3–105.5)	98.0 (73.8–132.3)	229.0 (149.0–564.0)	<.001
Total Bilirubin (T. bili) mg/dL^2^	0.5 (0.4–1.0)	0.6 (0.6–0.7)	1.5 (0.6–3.5)	<.001
Carcinoembryonic Antigen (CEA) mcg/L^3^	3.1 (1.1–4.5)	2.7 (1.2–3.6)	3.6 (1.9–6.3)	.476
Carbohydrate Antigen 19–9 (CA19-9) U/mL^4^	18.0 (9.5–50.0)	47.5 (26.0–199.3)	181.0 (122.0–342.0)	.042

Data are presented as the median (interquartile range) unless otherwise indicated. Continuous variables were compared by the Kruskal-Wallis test, while categorical variables were compared by the chi-squared test. There were 41 benign, 13 intrahepatic, and 22 extrahepatic Alkaline Phosphatase lab values available for analysis. There were 41 benign, 17 intrahepatic, and 24 extrahepatic Total Bilirubin lab values available for analysis. There were 10 benign, 9 intrahepatic, and 12 extrahepatic CEA lab values available for analysis. There were 12 benign, 14 intrahepatic, and 13 extrahepatic CA19-9 lab values available for analysis.

### Biliary CEACAM6 Levels

**Fig 1:**
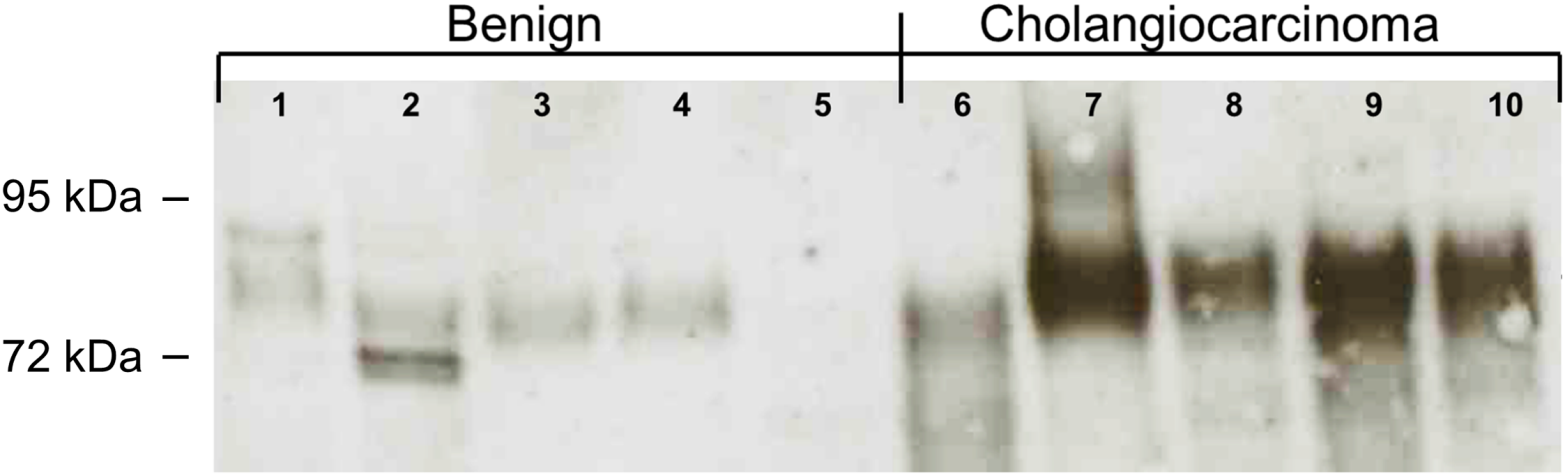
Qualitative Western Blot analysis of bile from patients with benign and malignant disease. The Western Blot analysis utilizes the detection antibody provided in the commercial ELISA kit used in these experiments. The 90kDa CEACAM6 is detected between the 95kDa and 72kDa protein ladder bands.

The concentration of biliary CEACAM6 as measured by immunosorbant assay is shown in Table 2. The median concentration of biliary CEACAM6 in patients with benign disease was significantly lower than that of patients with cholangiocarcinoma (7.5 [3.0–22.0] vs. 40.0 [15.0–260.8] ng/ml; p = <.001). This difference persisted when the benign cohort was compared to the intrahepatic (7.5 [3.0–22.0] vs. 25.0 [10.0–138.4] ng/ml; p = .051) and extrahepatic (7.5 [3.0–22.0] vs. 169.5 [25.5–498.5] ng/ml; p = <.001) cholangiocarcinoma subtypes. ELISA results were confirmed by Western analysis using a representative sampling of bile from patients with benign and malignant disease (Fig 1). The 90 kDa band corresponding to CEACAM6 was present in both cohorts but was more abundant in the bile of cholangiocarcinoma patients.

**Table 2:** Concentration of biliary CEACAM6 by immunosorbant assay.

Cohort	Median (IQR) [ng/mL]	*P* value
Benign (n = 42)	7.5 (3.0–22.0)	-
All Cholangiocarcinoma (n = 41)	40.0 (15.0–260.8)	< .001
Intrahepatic (n = 17)	25.0 (10.0–138.4)	.051
Extrahepatic (n = 24)	169.5 (25.5–498.5)	< .001

P value represents comparison to benign cohort utilizing the Mann-Whitney test. Data represents pooled results from two experiments.

### Clinical Predictability

**Fig 2:**
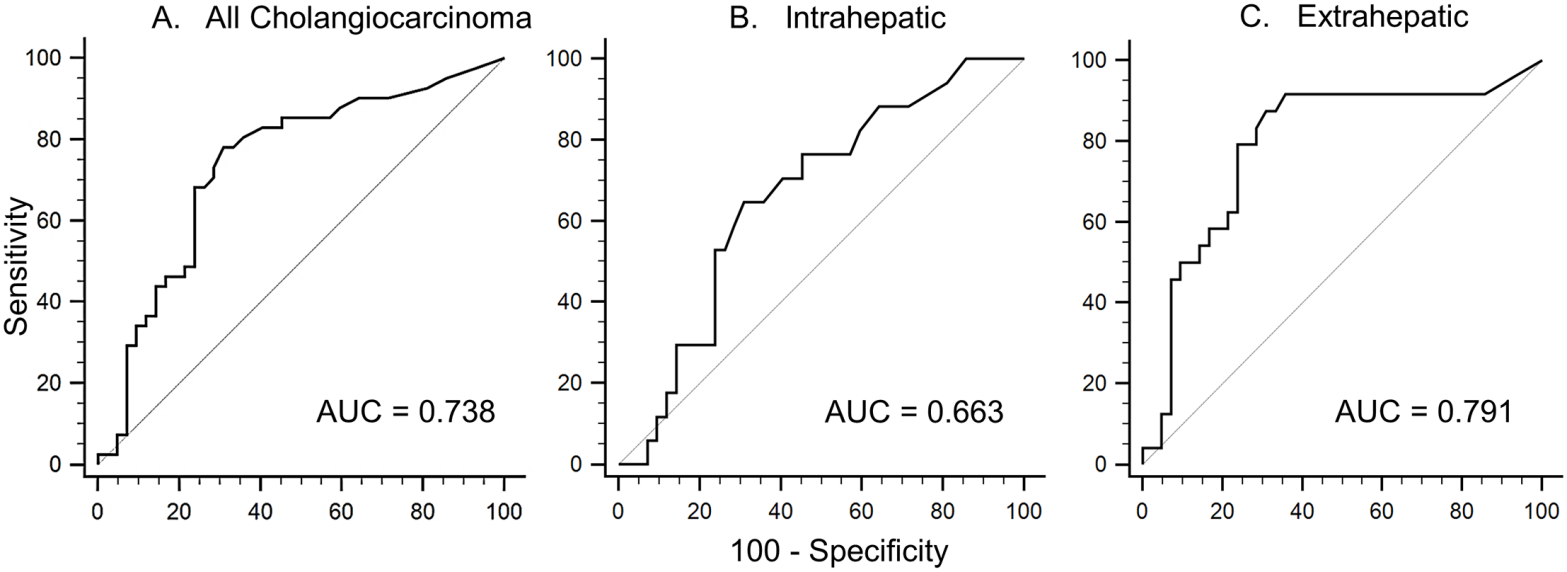
Receiver operating characteristic (ROC) curve analysis of CEACAM6 to evaluate presence of cholangiocarcinoma. The ROC analysis of all cholangiocarcinomas is depicted by graph A. with a resultant Area Under the Curve (AUC) of 0.738. Graphs B and C respectively illustrate the intrahepatic and extrahepatic subtypes with corresponding AUCs of 0.663 and 0.791.

To determine the predictive power for of CEACAM6 to diagnose cholangiocarcinoma, a receiver operating characteristic (ROC) curve was created (Fig 2). By using the area under of the curve (AUC) as a determinant of overall performance of the test, we found that biliary CEACAM6 could differentiate any cholangiocarcinoma (AUC = 0.738) or extrahepatic cholangiocarcinoma (AUC = 0.791) from benign disease. However, when CEACAM6 was used to differentiate intrahepatic tumor from benign disease, the AUC was 0.663. The optimal cut-off criterion for correctly identifying any cholangiocarcinoma was determined to be >14 ng/ml by Youden methodology. This cut-off is associated with a sensitivity of 87.5% and a specificity of 69.1% (Table 3).

**Table 3:** Predictive value of CEACAM6 in cholangiocarcinoma by various cut-off criterion.

Criterion	Sensitivity (95% CI)	Specificity (95% CI)	+LR	-LR
≥0	100.0 (85.8–100.0)	0 (0.0–8.4)	1.0	-
>0	95.8 (78.9–99.9)	7.1 (1.5–19.5)	1.0	0.6
*>14	87.5 (67.6–97.3)	69.1 (52.9–82.4)	2.8	0.2
>15	83.3 (62.6–95.3)	71.4 (55.4–84.3)	2.9	0.2
>22	79.2 (57.8–92.9)	76.2 (60.5–87.9)	3.3	0.3
>55	62.5 (40.6–81.2)	78.6 (63.2–89.7)	2.9	0.5
>80	58.3 (36.6–77.9)	83.3 (68.6–93.0)	3.5	0.5
>100	54.2 (32.8–74.4)	85.7 (71.5–94.6)	3.8	0.5
>168	50.0 (29.1–70.9)	90.5 (77.4–97.3)	5.3	0.6
>237	45.8 (25.6–67.2)	92.9 (80.5–98.5)	6.4	0.6
>825	12.5 (2.7–32.4)	95.2 (83.8–99.4)	2.6	0.9
>1145	4.17 (0.1–21.1)	100.0 (91.6–100.0)	-	1.0

* The cut-off of >14 ng/ml was associated with the highest Youden J index of 0.57.

### Factors potentially influencing biliary CEACAM6 concentration

We selected a number of potential dichotomous clinicopathological variables (age over 65 years, gender, biliary stent usage, bile salt concentration greater than the median of normal bile, or elevated CA19-9, alkaline phosphatase, and total bilirubin concentrations) that may impact biliary CEACAM6 secretion and compared measured levels (Table 4). We found differences in median CEACAM6 concentrations based on age (63.0 [15.8–167.5] vs. 9.5 [3.5–61.0]; p = 0.004), sex (55.0 [12.5–251.0] vs. 11.5 [3.0–28.0]; p = 0.006), stent presence (105.0 [28.0–449.0] vs. 11.0 [3.8–30.5]; p = <.001), elevated total bilirubin (166.0 [40.0–989.0] vs. 13.5 [4.0–132.0]; p = 0.003) and elevated alkaline phosphatase (215.0 [26.3–598.0] vs. 8.0 [3.0–24.0]; p = <.001).

**Table 4:** Differences in biliary CEACAM6 levels between potentially confounding patient characteristics.

Characteristic	Has characteristic	Lacks characteristic	*P* value
Age >65 years	63.0 (15.8–167.5)	9.5 (3.5–61.0)	0.004
Male sex	55.0 (12.5–251.0)	11.5 (3.0–28.0)	0.006
Stent in place	105.0 (28.0–449.0)	11.0 (3.8–30.5)	<.001
Bile salt <8.4 umol/mL	26.0 (5.0–246.0)	13.0 (5.0–110.0)	0.392
Carbohydrate Antigen 19–9 >100 U/mL	86.0 (23.3–617.3)	18.0 (8.0–80.0)	0.06
Total bilirubin >3.0 mg/dL	166.0 (40.0–989.0)	13.5 (4.0–132.0)	0.003
Alkaline phosphatase >140 IU/L	215.0 (26.3–598.0)	8.0 (3.0–24.0)	<.001

Data are presented as the median (interquartile range). Analysis performed by Mann-Whitney rank sum test.

We then performed linear regression analyses on these variables to determine association (Table 5). We found that CEACAM6 levels were weakly associated with age (R^2^ 0.090; p = 0.006), sex (R^2^ 0.081; p = 0.009), stent presence (R^2^ 0.198; p = <.001), total bilirubin (R^2^ 0.140; p = <.001), and alkaline phosphatase (R^2^ 0.211; p = <.001). However, only the presence of a biliary endoprosthesis (r_partial_ 0.351; p = 0.002) and alkaline phosphatase (r_partial_ 0.308; p = 0.007) were found by multiple regression analysis to be associated with elevated CEACAM6 concentrations. The R^2^ and *P* value of the overall multiple regression model were .308 and <.001 respectively.

**Table 5:** Regression analyses of clinicopathologic variables potentially associated with CEACAM6 levels.

		Linear Regression		Multiple Regression	
Characteristic	n	R^2^	*P* value	r _partial_	*P* value
Age	83	0.090	0.006	-	-
Male sex	83	0.081	0.009	-	-
Has stent	83	0.198	<.001	0.351	0.002
Total bilirubin level	82	0.140	<.001	-	-
Alkaline phosphatase level	76	0.211	<.001	0.308	0.007
Bile salt level	83	0.025	0.154		
Carbohydrate Antigen 19–9 level	39	0.000	0.979		

CEACAM6 levels were log transformed to achieve normal distribution. The number of patients with the given independent variable available for analysis is reported as (n). Variables found insignificant by linear regression were not included in the multiple regression model. Independent variables that were included in the multiple regression model but found to be unassociated are reported as “-”.

## Discussion

Currently, there is no reliable method for screening individuals at high risk for developing cholangiocarcinoma, a disease with few options for treatment in advanced stages. Early detection and complete surgical resection offer the only chance for cure. Imaging studies are often inaccurate until the lesion is larger than 20 mm and will frequently miss non-mass forming subtypes of cholangiocarcinoma (periductal infiltrating or nodular sclerosing).[28] Endoscopic evaluation of the biliary system with cytologic brushings and subsequent analysis by fluorescence in situ hybridization has reported sensitivities of 38–58%.[29–31] This poor predictability could be due to the highly desmoplastic features of cholangiocarcinoma limiting adequate sampling. The most commonly used tumor marker has been serum CA19-9, a test associated with a variable sensitivity and specificity. Bile based biomarkers have been investigated as a potentially more sensitive alternative due to direct contact with tumor. Previous studies have demonstrated that biliary IGF-1 was 15–20 fold higher in patients with extrahepatic cholangiocarcinoma compared to levels in patients with pancreatic carcinoma or benign disease with an AUC of 1.[32] CEACAM6 overpression in pancreatic cancer is known to increase IGF-1 mediated cellular invasiveness through an Akt and Src-dependent pathway. Given the elevated levels of IGF in the bile of cholangiocarcinoma, this may be a potential mechanism for CEACAM6 carcinogenesis.[33] Minichromosome replication proteins (MCM) have been investigated as potential biomarkers with biliary MCM5 levels being more sensitive than brush cytology (66% vs 20%) for the diagnosis of cholangiocarcinoma.[34] Biliary Mac-2BP levels were found to be as good as serum CA 19–9 with an AUC of 0.70 to detect biliary cancer from PSC and other benign biliary disease.[35] A study investigating the use of capillary electrophoresis mass spectrometry to screen for specific peptide markers in urine has shown potential in differentiating benign from malignant biliary processes with an AUC of 0.87.[36] However these initial results in both studies remain to be validated in larger cohorts of patients. The present study demonstrates that biliary CEACAM6 may be potentially more sensitive than current serological, radiographic, or cytological analyses for cholangiocarcinoma.

Our cohorts were demographically similar with the exception that those with cholangiocarcinoma were older by a decade. This is likely due to the inherent bias of a younger population with benign disease compared with the increased incidence of cancer in older patients. Those with EHCC had laboratory evidence of biliary obstruction with decreased bile salt concentrations, elevated serum alkaline phosphatase, and increased total bilirubin levels; which is in agreement with the late onset presentation and high incidence of biliary obstruction in these patients. Endoscopic retrograde cholangiopancreatography is often performed after initial assessment for confirmation of stricture, placement of a biliary stent, and biopsy. This is reflected in our study population with a significantly higher percentage of stents present in the extrahepatic cohort compared to those with IHCC (83% vs. 6%).

Median biliary CEACAM6 levels by ELISA were significantly higher in cholangiocarcinoma patients (40.0 vs. 7.5 ng/ml). When ROC curves were created to determine the ability of CEACAM6 to predict disease we found that it was slightly more diagnostic of EHCC (AUC = 0.791) than all cholangiocarcinomas combined (AUC = 0.738). The associated sensitivity of 87.5% is much better than the 53% initially published for serum CA19-9 in the general population, while the specificities were similar (69.1% vs. 76.0% respectively).[37] Previous studies have shown that CEACAM6 is overexpressed in IHCC tumor tissue, and this is reflected by higher biliary CEACAM6 levels (25.0 vs. 7.5 ng/ml for benign disease, p = .051). However, biliary CEACAM6 levels in EHCC were substantially higher than in benign disease (7.5 vs. 169.5 ng/ml; p = <.001) and IHCC (25.0 vs. 169.5 ng/ml; p = .018). This difference in CEACAM6 biliary levels between EHCC and IHCC may be attributable to inherent differences in protein expression and/or excretion, contact time between bile and tumor cells, and/or some degree of biliary obstruction.

Unlike serum, the concentration of solutes in bile can change dramatically based on degree and location of ductal obstruction. In addition, protein abundance and subsequent detection may be affected by the presence of bile pigments or salts, reflected in the present study by the variable concentrations of measurable bile salts in the different cohorts. As expected, the median bile salt concentration was most dilute (i.e. lowest) in the EHCC subgroup (5 μmol/mL) likely due to the large proportion of patients with biliary obstruction (83% requiring a stent).

To investigate potential confounders influencing CEACAM6 levels we identified four potential populations that could be expected to have different degrees of secretion: 1) Biliary obstruction (Elevated total bilirubin, elevated alkaline phosphatase, stented, or bile salt concentration below benign median) 2) Advanced age (age over 65) 3) Gender 4) Elevation in standard tumor marker (CA19-9 elevation). Comparison of CEACAM6 concentrations between these cohorts found differences in all but those with elevated CA19-9 or low bile salts. Linear regression was then performed on these same variables and identified associations between all tested factors except CA19-9 and bile salt levels. The lack of association with CA19-9 levels could be either due to the poor predictability of this marker for cholangiocarcinoma or because we only had these values on 47% of our test population. Subsequent multiple regression analysis found only alkaline phosphatase concentration and stent presence were associated with biliary CEACAM6 levels. These findings suggests that the dilutional effect of obstruction, as measured by bile salt and total bilirubin concentrations, does not have as a strong an impact on CEACAM6 levels as the inflammatory component as inferred by the association with elevated alkaline phosphatase and stent usage.

The presence of a stent could potentially affect the level of biliary CEACAM6 by either mechanically disrupting the epithelial lining and thereby increasing the shedding of CEACAM6 or by inducing inflammation and recruiting neutrophils that naturally express CEACAM6 as an adhesion molecule into the duct. It is likely that the mere presence of a stent is not the sole driver of CEACAM6 concentration as levels in IHCC were higher than in patients with benign disease with a similar low proportion of biliary stents (12% vs 6%). However, this significant confounder must be addressed in subsequent studies before CEACAM6 can be recommended for clinical use.

The results from this study suggest biliary CEACAM6 may be useful in differentiating cholangiocarcinoma from benign biliary disease. We feel that this test would be most useful for screening high risk individuals (e.g. primary sclerosing cholangitis patients) to identify malignant changes early. Although no there is no current consensus on surveillance, many patients with primary sclerosing cholangitis do undergo annual screening with either ultrasound or magnetic resonance cholangiopancreatography to evaluate for worrisome strictures. If structuring is found an endoscopic retrograde cholangiopancreatography usually follows with brushings of the duct to diagnosis malignancy. CEACAM6 may be useful by either being a more accurate confirmatory test than those currently available or as a screening test with endoscopic evaluation at set intervals. Future studies are needed to determine if CEACAM6 is differentially expressed in other biofluids such as serum, urine, or stool and/or could be collected in a less invasive manner.

The authors recognize a number of limitations in this study, chief among these were the high percentage of extrahepatic cholangiocarcinoma with history of biliary obstruction and concurrent endoprostheses. Additionally, the lack of corresponding CA19-9 in all patients made the evaluation of the combinatory diagnostic ability of CEACAM6 and CA19-9 impossible. It is possible that the predictive abilities of serum CA19-9 and serum or biliary CEACAM6 may be additive. Validation of these results in a separate cohort must be performed before clinical use can be recommended.

## Conclusion

Biliary CEACAM6 can identify patients with extrahepatic cholangiocarcinoma with a high degree of sensitivity and should be investigated further as a potential screening tool in high-risk populations.

## Supporting Information

S1 Table Pathologic staging. (DOCX)
