# Supplementary material for: The Role of Biliary Carcinoembryonic Antigen-Related Cellular Adhesion Molecule 6 (CEACAM6) as a Biomarker in Cholangiocarcinoma
Source: PLoS One. 2016 Mar 14;11(3):e0150195. doi: 10.1371/journal.pone.0150195 (PMC4790932; doi:10.1371/journal.pone.0150195)
Supplement: S1 Table — (DOCX) [file pone.0150195.s001.docx]

**S1 Table:** Pathologic Staging

| Patient ID | Tumor Location | Gender | Age (yrs) | Had Biliary Stent | Serum CA 19-9 (U/ml) | Biliary CEACAM6 (ng/ml) | Resected | Pathologic Stage |
| --- | --- | --- | --- | --- | --- | --- | --- | --- |
| 1 | Hilar | M | 63 | Y | 366 | 1007 | Y | 1 |
| 2 | Intrahepatic | M | 79 | N | 53 | 35 | Y | 2 |
| 3 | Distal | M | 64 | Y | 130 | 246 | Y | 1b |
| 4 | Intrahepatic | M | 73 | N | * | 10 | Y | 3 |
| 5 | Intrahepatic | F | 48 | N | 3.7 | 12 | Y | 2 |
| 6 | Distal | F | 59 | Y | 128 | 1 | N |  |
| 7 | Distal | M | 43 | Y | 1037 | 24 | N |  |
| 8 | Distal | F | 58 | Y | * | 989 | Y | 1b |
| 9 | Intrahepatic | M | 40 | N | 850 | 5 | N | 4a |
| 10 | Intrahepatic | F | 55 | N | 71 | 15 | N |  |
| 11 | Distal | F | 89 | Y | * | 27 | Y | 2b |
| 12 | Intrahepatic | M | 62 | Y | 1 | 225 | Y | 3 |
| 13 | Distal | M | 45 | N | 104 | 548 | Y | 3 |
| 14 | Intrahepatic | F | 63 | N | 26 | 6 | Y | 1 |
| 15 | Intrahepatic | F | 85 | N | 42 | 2 | Y | 4a |
| 16 | Hilar | M | 66 | Y | * | 449 | N |  |
| 17 | Distal | F | 57 | Y | * | 788 | Y | 2a |
| 18 | Distal | M | 77 | Y | * | 124 | Y | 2a |
| 19 | Distal | M | 77 | N | 13 | 364 | Y | 1a |
| 20 | Hilar | F | 43 | Y | 2 | 748 | Y | 3b |
| 21 | Intrahepatic | M | 67 | N | 9128 | 21 | Y | 1 |
| 22 | Intrahepatic | F | 66 | N | 302 | 259 | Y | 2 |
| 23 | Intrahepatic | F | 65 | N | 26 | 3 | N |  |
| 24 | Hilar | M | 56 | Y | * | 383 | Y | * |
| 25 | Hilar | M | 76 | Y | 334 | 40 | Y | * |
| 26 | Hilar | M | 71 | Y | 167 | 86 | Y | * |
| 27 | Distal | M | 64 | Y | * | 13 | Y | * |
| 28 | Hilar | M | 73 | Y | * | 15 | Y | * |
| 29 | Hilar | F | 84 | Y | 181 | 63 | Y | 3a |
| 30 | Intrahepatic | F | 65 | N | * | 25 | Y | 3 |
| 31 | Hilar | F | 69 | Y | 1187 | 1296 | N |  |
| 32 | Intrahepatic | M | 61 | N | 37 | 40 | N |  |
| 33 | Hilar | M | 57 | N | * | 266 | Y | 3b |
| 34 | Intrahepatic | F | 77 | N | * | 144 | Y | 3a |
| 35 | Intrahepatic | M | 63 | N | 25 | 29 | Y | * |
| 36 | Distal | F | 67 | N | * | 18 | Y | * |
| 37 | Intrahepatic | F | 74 | N | 79 | 166 | N |  |
| 38 | Intrahepatic | M | 74 | N | 199.3 | 140 | Y | 1 |
| 39 | Distal | M | 75 | Y | * | 215 | Y | 2b |
| 40 | Hilar | F | 79 | Y | 234 | 0 | Y | 4a |
| 41 | Distal | F | 66 | Y | 185.8 | 28 | Y | 2b |

* Data unavailable

Extrahepatic tumors include both hilar and distal locations.

Staging based on American Joint Committee on Cancer, 7th edition criteria.
